# Effect of Extensive Solar Ultra-Violet Irradiation on the Durability of High-Density Polyethylene- and Polypropylene-Based Wood–Plastic Composites

**DOI:** 10.3390/polym17010074

**Published:** 2024-12-30

**Authors:** Mohammad N. Siddiqui, Halim H. Redhwi, Anthony L. Andrady, Sarfaraz A. Furquan, Syed Hussain

**Affiliations:** 1 Chemistry Department and IRC-Refining and Advanced Chemicals, King Fahd University of Petroleum & Minerals, Dhahran 31261, Saudi Arabia; 2 Chemical Engineering Department and IRC-Refining and Advanced Chemicals, King Fahd University of Petroleum & Minerals, Dhahran 31261, Saudi Arabia; hhamid@kfupm.edu.sa; 3 Department of Chemical and Biomolecular Engineering, North Carolina State University, Raleigh, NC 27607, USA; alandrad@ncsu.edu; 4 Mechanical Engineering Department, King Fahd University of Petroleum & Minerals, Dhahran 31261, Saudi Arabia; sfurquan@kfupm.edu.sa; 5 Dhahran Techno-Valley, King Fahd University of Petroleum & Minerals, Dhahran 31261, Saudi Arabia; syedhussain@kfupm.edu.sa

**Keywords:** wood–plastic composites, weatherability, HDPE, PP

## Abstract

The natural and laboratory-accelerated weathering of wood–plastic composites (WPCs) based on high-density polyethylene (HDPE) and polypropylene (PP) plastics was investigated in this study. Injection molded samples of WPCs with different loadings of wood fiber ranging from 0 to 36 wt.% of wood were subjected to laboratory-accelerated weathering and natural weathering. The integrity of samples weathered to different extents was tested using a standard tensile test and surface hardness test to investigate the dependence of these properties on the duration of weathering exposure. Tensile data were used to identify the loading of wood fibers in either plastic matrix that afforded superior ultra-violet (UV) stability. Tensile measurements under uniaxial strain yielded average values of tensile strength (TS), low-extension modulus (E), and elongation at break (EB). Both natural weathering outdoors and accelerated weathering in the laboratory showed that the TS and EB decreased while the E increased with the duration of exposure for all samples tested. The change in the average TS of composites with the duration of exposure offers valuable insights. The correlation between the tensile and hardness data for the WPC samples was explored. After naturally weathering at two exposure sites, the hardness of the WPCs was found to decrease between 8% to 12.5%, depending on the composition and exposure location parameters. Furthermore, no marked difference in performance with increasing wood fiber beyond 18 wt.% was observed. WPCs can be a key parameter in environmental sustainability by being used in the building and packaging industries, which reduces carbon emissions and waste generation.

## 1. Introduction

A clear trend of the materials used in the building and packaging industries is towards increased environmental sustainability. This emphasis typically demands lowering energy costs, reducing carbon emissions, conserving non-renewable materials such as fossil fuels, and reducing waste generation in building construction. Using plastics in place of wood not only depletes non-renewable fossil-fuel resources but also creates an increasing stream of non-degradable post-consumer plastic waste that must be managed. The building industry typically has a high energy demand. In 2020, 30% of the total global energy expenditure and 26% of global carbon emissions that contributed to global warming were attributed to the building industry [1]. While significant gains in the sustainability of plastics can be achieved by material recycling [2], which conserves fossil-fuel raw material and enables a circular economy [3], effectively managing municipal and especially unmanaged solid waste still poses a significant challenge in most regions of the world. The global production of plastic resins is slated to increase over the near term and the ensuing impacts of plastic waste will exacerbate in the future, with the expected doubling of buildings in the world by 2060 adding over 230 billion sq.m. (relative to 2017 data) of built space [4].

Wood is a proven building material heavily used in the construction industry. It is a heterogeneous, cellular, and anisotropic material composed of cellulose (40–50%) and hemicellulose (15–25%) fibers with lignin (15–30%) [5]. It has always been regarded as a low-cost, sustainable building material with a relatively low carbon footprint relative to the material used in buildings such as concrete, metals, glass, or plastics. Using more wood, which is both renewable and recyclable, in buildings increases their environmental sustainability. However, wood as a building material has the drawback of limited durability in outdoor use, especially in decking, fencing, and outdoor furniture. Lignin, being a good chromophore, undergoes facile degradation under solar UV exposure, resulting in surface degradation [6,7], and the increased hydrophilicity of the affected surface facilitates biodegradation [8]. The effects of common biological degrader species such as white-rot and brown-rot fungi are well known. Some of these limitations can be overcome by using WPCs, where wood fibers are used as a filler in a plastic matrix, in place of wood in building construction.

The development of WPCs dates to the 1970s but they have been popularly used as a building material in North America since the 1990s [9]. Natural wood fibers are significantly lower in density (1.15–1.50 g/cm^3^) than the glass fibers (2.4 g/cm^3^) commonly used in fiber-reinforced composites. Consequently, WPCs provided a significant weight reduction of the products, leading to reduced transportation costs. WPCs have the advantage of a high strength-to-weight ratio and ease of manufacture [10] associated with plastic materials. The design freedom afforded by WPCs is comparable to that afforded by plastics in that the material can be formed into single-piece building products, saving considerable labor, energy, and carbon emissions [11]. However, wood fibers have a lower elastic modulus than glass or carbon fiber [12]. Incorporating wood fiber into a thermoplastic matrix generally affords WPCs that combine the appearance and rigidity of wood and the toughness and processability of plastics in a single material. The material is used primarily in exterior furniture, decking, playground equipment, fencing, and roofing [13,14]. The wood fibers in the composite can still degrade on environmental exposure but are relatively more durable than wood. It also has the sustainability advantages of using agricultural waste such as straw [15] or wood flour waste [16,17] as well as recycled waste plastics [18] in its composition [19].

WPCs are generally manufactured in a two-step process. A thermoplastic, such as HDPE, low-density polyethylene (LDPE), or PP, is blended with fine wood fibers of sizes ranging from 20 mesh to 60 mesh in a compounding extruder and pelletized as a masterbatch [20], usually in a continuous process in commercial production [21,22]. The compound also often includes compatibilizers, lubricants, flame retardants, and colorants. The compatibilizer, usually a maleated polyolefin, allows the intimate mixing of the hydrophilic wood with the hydrophobic polymer while the lubricant yields better surface characteristics in extruded products. The masterbatch is mixed with thermoplastic at the required let-down ratio and extruded into the product [23]. As most wood fibers tend to degrade above 210 °C (410 °F), processing temperatures must be controlled below this limit 17. However, the common thermoplastics used in WPC applications, polyethylene (PE), poly (vinyl chloride) (PVC), and PP [24], can be processed below these critical temperatures. Even with exhaustively dried wood fibers, a weak interface obtains poor stress transfer through the composite, negatively impacting its mechanical properties [25]. To overcome these shortcomings, wood fibers can either be treated to render them hydrophobic [26] or a compatibilizer such as maleated polyolefin can be used in the compound [27].

### Weathering of WPCs

The most important limitation of WPCs is the deterioration of mechanical properties and the durability of WPC materials against weathering [28,29]. This is primarily a consequence of the photostability of the wood fiber fraction in the composite. Solar UV radiation, especially the UV-B range of the spectrum, is well known for its deleterious effect on both wood [30] and plastic [31] components. The breakdown of lignin generates chromophoric water-soluble reaction products, such as carbonyls, carboxylic acids, quinones, and hydroperoxy radicals [32]. WPCs based on PP and HDPE are well known for their sensitivity to UV radiation [33,34,35,36,37].

The exposure of WPCs to solar UV radiation results in the surface yellowing of the wood, affecting its aesthetic appeal. Treating the wood fibers used in WPCs chemically to make them less chromophoric is a reasonable approach to control degradation. For instance, Darabi et al. methylated or acetylated the lignin moieties in wood fibers to effectively control the yellowing of wood–HDPE composites [38]. Solar UV-induced degradation is measured as a decrease in average flexural properties, using conventional UV absorbers [39]. Recent work found the incorporation of 1–2 wt.% of titanium dioxide to protect WPCs against weakening under exposure to solar-simulated UV radiation was effective [40]. However, whether these approaches would work under desert exposure conditions that combine high UV levels and high sample temperatures is unknown.

**Figure 1:**
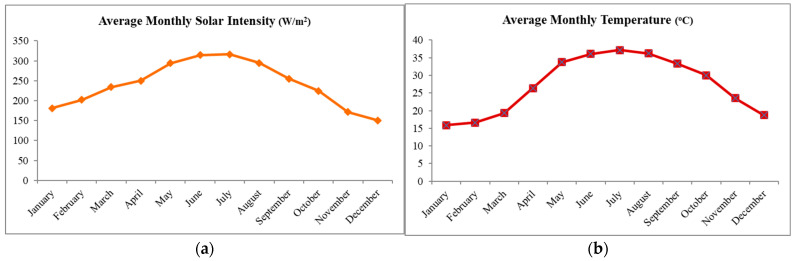
(**a**) Average annual solar intensity variation and (**b**) average temperature variation at the desert exposure site in Dhahran, Saudi Arabia.

The present study is intended to study the weathering of two WPC formulations based on either HDPE or PP as the polymer matrix and commercial wood fiber as the filler, compounded at four different weight percentages of the wood component, with maleated polyolefin used as the compatibilizer. Over 70 percent of WPC production is used in buildings, with polyethylene (PE) and PP being the most-used plastics in their composition [41]. Both natural and accelerated weathering exposures were employed in the present study. Part I of this paper [42] addressed the mechanical characteristics of PP- and PE-based wood–plastic composites and the present part 2 of the paper is a research effort on the weatherability of the same WPCs exposed to outdoor weathering under harsh desert exposure conditions, as shown in Figure 1.

## 2. Materials and Methods

The wood fiber was obtained from Jelu-Werk (Rosenberg, Germany) as masterbatches with 50% wt. wood fiber (spruce and fir) content in either PP (Grade PP-H50-500-14) or HDPE (Grade HDPE-H50-500-09) compounds. The two base resins used are injection moldable grades of HDPE and PP with no added UV stabilizers. The bulk density of the masterbatches was 550 g/L and their melt flow index (190 °C/21.6 kg) was 33 and 60 g/10 min, according to DIN ES ISO 1133 for the PE and PP masterbatches, respectively. All compounds had a maleated polypropylene bonding agent (2 wt.%) and 1–2% of a lubricant such as ethylene bis-stearamide (EBS) or zinc stearate. The masterbatch compounds were mixed with the appropriate proportion of either PP (Hival 2620) or HDPE (Hival 521054) to obtain the requisite weight fractions of wood between 18% and 50%. The masterbatches were dried in the hopper for 5 h at 80 °C before injection molding. Moldings were carried out with a screw temperature of 190 °C and mold temperature of 80 °C with a screw speed maintained as low as possible to avoid overheating the wood fiber. Standard ASTM Type I dumbbell test pieces were molded and kept refrigerated in the dark.

The natural weathering of the samples was carried out under desert exposure conditions (at an exposure site in Dhahran, Saudi Arabia). The harsh weathering conditions involved are reflected in the plot of average monthly temperature and solar UV flux at the location during the year, as shown in Figure 1. Outdoors, factors such as humidity and atmospheric pollutants can affect the rate of weathering of WPCs even though solar UV radiation is the predominant factor. Accelerated weathering in the laboratory was carried out in a Q-SUN Xenon test chamber model Q-Sun (Xe-3) at an irradiance of 0.4 W/m^2^/nm measured at 340 nm wavelength equipped with a daylight filter. Continuous illumination at 70 °C was used without any dark period or water spray.

Tensile testing was done on five sample replicates generally according to ASTM D 638 using an Instron Universal Testing Machine, Model 3367, at room temperature (20 to 22 °C) at a strain rate of 10 mm/min strain rate. Load displacement data were recorded digitally using a computer attached to the machine. Hardness measurements on the Shore D scale were done using the Durometer-Digi Test of Bareiss Germany according to the ASTM D 2240 standard. An indenter cone with an angle of 300 and a contact pressure load of 5100 g for 3 s dwell time was used, with the depth of penetration set equal to 2.5 mm.

Morphological analysis of the microstructure was performed using a JEOL JSM-6064LV Scanning Electron Microscope. Morphological images of the external surface of the samples were captured at an accelerating voltage of 15 kV with various magnifications. Thermal transition temperatures were assessed using a Mettler Toledo DSC 822 Differential Scanning Calorimeter (DSC). Samples were heated from ambient temperature to 200 °C at a rate of 10 °C/min under an argon gas atmosphere. The melting temperature was determined as the peak temperature and the enthalpy of melting was calculated from the peak area after baseline adjustment.

Fourier transform infrared (FTIR) spectroscopy was employed to analyze the molecular structures of wood and plastics, as it provides a distinctive “fingerprint” for the chemical bonds of the composites through molecular vibrations. Reflectance FTIR was utilized for thicker samples and spectra were recorded using a Perkin Elmer 16F PC FT-IR spectrophotometer equipped with Spectrum V 2.00 software.

The molecular weights of the PP and HDPE polymers were measured using Gel Permeation Chromatography (GPC), a size-exclusion chromatography technique that separates polymer particles based on molecular size. This method evaluates molecular weight metrics such as the number average molecular weight (Mn), weight average molecular weight (Mw), size average molecular weight (Mz), and the polydispersity index (PDI), which varied with polymer degradation. Calibration was conducted using polystyrene standards with PDI values below 1.2. GPC measurements were performed at 160 °C in 1,2,4-trichlorobenzene (TCB) with a flow rate of 1.0 mL/min and an injection volume of 200 µL, using the Viscotek HT-GPC 350A system.

## 3. Results

### 3.1. Tensile Properties

**Figure 2:**
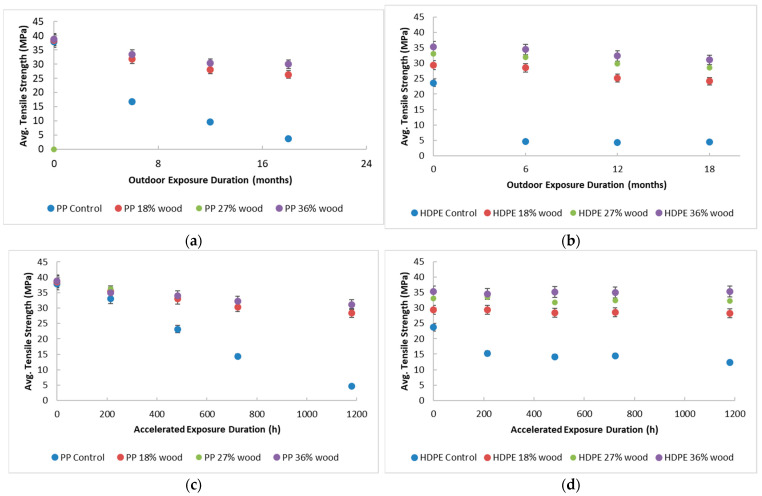
Average tensile strength data for WPCs based on HDPE and PP. (**a**) Outdoor exposure for PP composites; (**b**) outdoor exposure for HDPE composites; (**c**) accelerated exposure for PP composites; and (**d**) accelerated exposure for HDPE composites.

The rate of deterioration in the tensile properties of the composites under either outdoor or accelerated weathering exposure is a good indicator of their durability. Tensile measurements under uniaxial strain yield average values of TS, E, and EB. The average EB is particularly sensitive to weathering and its value drops drastically even during early weathering in the present exposures. This is likely a result of adhesive failure at the wood/plastic interphase dominating sample rupture. Both natural weathering outdoors and accelerated weathering in the laboratory generally show the tensile strength and the elongation at break decreasing with the duration of exposure while the low-extension modulus increases with the duration of exposure for all samples tested (see Table 1). The change in the average TS of composites with the duration of exposure offers valuable insights (see Figure 2).

The average TS decreases approximately linearly with the duration of weathering with exposure in the control samples with no wood fiber and the rate of decrease is faster in PP relative to HDPE. This is an expected consequence of the repeat unit chemical structure of PP, which makes it relatively more photolabile [42,43]. The presence of wood fibers at all weight fractions used in the present study also shows a similar near-linear decrease in the percentage loss of average TS, but the rate of loss is much lower, suggesting significant UV stabilization. This result is generally consistent with previous work on the weathering of PP-based [35,44] and HDPE-based [45] WPCs, despite the present exposures being much harsher in terms of UV flux and sample temperatures. The percentage of reduction in the average TS at a given duration of exposure depends not only on the loading of the wood fibers but also on their type, how well they are dispersed in the matrix, and the compatibilizer used [46]. It is difficult to compare data across different accelerated exposure studies because of these variables.

For instance, Stark et al., working with HDPE/Pine (50%) composites, found a 32–34% decrease in the average TS in 3000 h. of weatherometer exposure, while the present WPCs reached the same in 1180 h of weatherometer and 12 months of outdoor exposure. The average TS data in Figure 2 (which shows data for the WPCs) suggest a significant stabilization of composites containing wood fiber, with improvement in durability with wood content in the case of the HDPE-based composites. This is true of the present WPCs based on PP as well but their rate of loss in average TS is faster than for the HDPE-based WPCs’ mechanical integrity of the composites. Furthermore, no marked difference in performance with increasing wood fiber beyond 18 wt.% is observed. That relatively low loading of 18 wt.% wood provides good photostability and is therefore a valuable finding in designing WPCs for outdoor use.

Both the light shielding by fibers and the UV-stabilizing action of phenolic lignin compounds in wood retard photo-thermal degradation [47] and are responsible for the stabilization effect. With UV shielding, the overlapping of wood fibers at the concentrations used in the present composites would make it difficult to observe any dependence of weatherability on wood content. As the exposed WPC samples reached high surface temperatures, especially in the natural weathering environment, concurrent thermal oxidation would also contribute to the observed decrease in tensile strength. Laboratory studies on WPCs of PVC/poplar wood show the effect of temperature alone on tensile strength [48].

In both the control samples and the WPC samples, the average E increases with the duration of exposure, suggesting extensive crosslinking during exposure. Concurrent chain scission and crosslinking are known to occur in polyolefin photo-oxidation [49], which is a free-radical reaction [50], and under present exposure conditions crosslinking is predominant, leading to increased moduli. Furthermore, the increased crystallinity of the polymer component during photodegradation may also have contributed to this increase.

A set of samples containing 18 wt% wood was also exposed for 330 MJ/m^2^ {12,686 MJ/m^2^ of total energy} in a UA-EMMA (Atlas) to determine their behavior under extended long-term exposure. The UA-EMMA employs twenty focusing mirrors arranged as parabolic facets to variably concentrate UV energy on a target area of approximately 15 cm × 91 cm (6 inches × 36 inches) that is protected from overheating. It is used as an accelerated exposure to simulate long-term weathering. Unexpectedly, the samples exposed retained their integrity and their tensile properties could be measured (see Table 1) and their average tensile strength was found to be approximately the same as for samples exposed in the laboratory weatherometer for 743 kJ/sq.m (orders of magnitude lower than the exposure in the UA-EMMA). At the very high light intensities in the UA-EMMA, the degradation of the WPCs likely proceeds via reaction pathways that are very different from those obtained under natural solar or weatherometer irradiation. The data for the high-intensity exposures were inconsistent with the laboratory data from weatherometer exposures and the samples were not further analyzed.

### 3.2. Hardness Properties

With thick samples such as those used here, photo-thermal degradation is likely localized to a surface layer. This is known to be the case for both wood [51] and polyolefins [52]. With the polymers, the photooxidation is diffusion-controlled in thick sections of the material. Unlike the tensile measurements that assess the bulk of the sample, hardness testing that tests the surface characteristics is useful in assessing weathering [53]. Unlike flexure and tensile testing, hardness is rarely used to monitor the weathering of WPCs. However, hardness, being a surface characteristic, is expected to be more sensitive to weathering-related changes.

Including wood fiber filler in a thermoplastic matrix generally affects the hardness of the material [54]. The Shore D hardness of the various WPCs, based on either HDPE or PP, as well as the control samples, decreases with the duration of weathering exposure. With the data for accelerated weathering in the laboratory, the percent decrease is linear with the duration of exposure. Table 2 shows the changes in the average Shore D hardness of samples at different durations of exposure. The measurements were carried out on the surface exposed to light. The observed decrease in hardness with duration of exposure is consistent with previous reported studies [53,55]. For instance, Srivabut et al. (2023) [56] studied the natural weathering of PP/rubberwood composites with 0, 25, and 50 wt. percent wood fiber with a maleated polypropylene compatibilizer. After naturally weathering at two exposure sites, the hardness of the WPCs was found to decrease between 8% to 12.5% depending on the composition and exposure location parameters. The present PP-based WPCs also lost approximately 8% of their original hardness even though they were subjected to much harsher weathering conditions.

The wood fiber filler improved the retention of surface hardness in both HDPE and PP composites relative to the control plastic material. In common with the tensile strength data, changes in hardness also show HDPE composites to be better stabilized relative to those of PP. However, in contrast to the tensile test data, the hardness data show that different loadings of wood fiber in the HDPE-based WPCs make a significant difference in the rate of loss of surface hardness in accelerated weathering studies. However, the natural weathering data for PP do not show such a difference. The mechanisms that result in changes in hardness for PP might be different in natural exposures versus laboratory-accelerated exposure scenarios. One such variable is the abrasion of the WPCs’ surface by wind-borne sand in desert environments. Another is the effect of humidity (or water) on the composites. The wood fiber in WPCs absorbs humidity either through exposed fiber ends at the surface or from small amounts of water absorbed by the plastic matrix.

### 3.3. Tensile Strength Versus Surface Hardness

**Figure 3:**
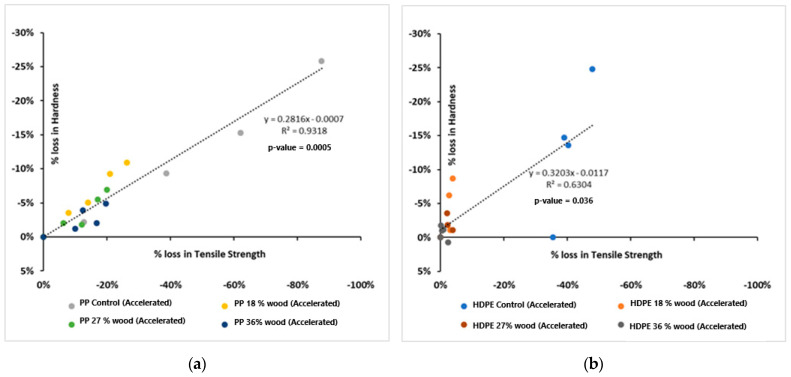
Percentage loss in bulk tensile strength versus percentage loss in surface hardness for WPCs based on HDPE and PP. (**a**) Accelerated exposure for PP composites; (**b**) accelerated exposure for HDPE composites.

An interesting observation in the data is that loss in changes in the two properties of bulk tensile strength and surface hardness behave similarly on weathering exposures. Figure 3 shows how the two sets of data are related for all PP-based WPCs tested in the study, where a high correlation (R^2^ = 0.93) between the data is apparent. However, the same plot attempted for HDPE-based composites shows a poor correlation (R^2^ = 0.63). With PP-based composites, this correlation allows the relatively faster hardness tests using smaller samples to be employed to test durability in place of tensile property measurements.

Hardness and tensile strength are both important properties of materials, but they describe different aspects of material behavior. In many materials, there is a general correlation between hardness and tensile strength. Typically, harder materials tend to have higher tensile strength. This is because both properties are influenced by the material’s microstructure and bonding. The relationship can vary significantly among different materials. For example, some polymers may have high toughness but low hardness, while certain ceramics may be very hard but brittle, exhibiting low tensile strength. While hardness and tensile strength are related and often influence each other, they represent distinct properties.

### 3.4. FTIR Characterization

**Figure 4:**
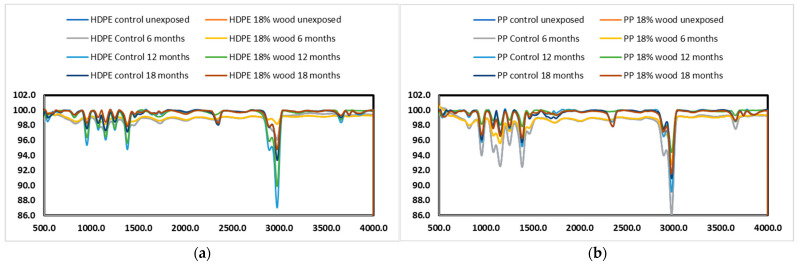
Overlay of FT-IR spectra of naturally exposed samples of WPCs based on HDPE and PP. (**a**) Outdoor-exposed HDPE composites with 0 and 18% wood filler; (**b**) outdoor-exposed PP composites with 0 and 18% wood filler.

Combining the wood fiber into a polymer matrix can be tracked using Fourier Transform Infrared Spectroscopy in the Attenuated Total Reflection (FTIR-ATR) mode. The spectra of dry composites reveal a characteristic hydroxyl (-OH) absorption peak, attributed to cellulose and hemicellulose, typically observed near 3500 cm⁻^1^. The same absorption band is also present in both HDPE and PP polymers within the specific wavelength range. Moreover, the intensity of the band increases proportionally with the wood fiber content, as illustrated in the inset of Figure 4. Incorporating wood fibers does not significantly affect the polymers’ melting properties since the fibers themselves do not undergo phase transitions during heating.

### 3.5. Differential Scanning Calorimetry (DSC)

**Figure 5:**
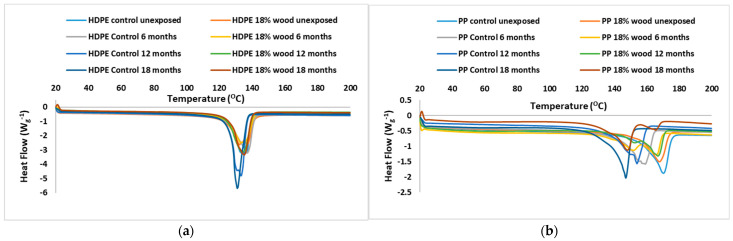
Differential Scanning Calorimetry of naturally exposed samples of WPCs based on HDPE and PP. (**a**) Outdoor-exposed HDPE composites with 0 and 18% wood filler; (**b**) outdoor-exposed PP composites with 0 and 18% wood filler.

Differential Scanning Calorimetry (DSC) analysis of the polymer–wood composites afforded the analysis of a reciprocal relationship between heat flow and wood fiber contents. Figure 5 shows that the heat flow decreases with increasing wood fiber content as anticipated, while the melting transition temperature of the polymer fraction remains largely unaffected by the presence of wood fibers. Changes observed in both FTIR and thermal measurements of the composites qualitatively correlate with the wood fiber content, implying that the wood fibers and polymer matrix are well integrated within the composite matrix. The spectra do not display a dominance of spectral bands associated with either wood fiber- or polymer-rich regions, indicating a uniform distribution of components in the composites.

The DSC curves of WPCs based on HDPE and pure HDPE indicate that the peak height and area clearly depend on the HDPE percentage. These parameters decrease with HDPE content. This means that the observed peak is totally connected with the polymer phase transition.

### 3.6. Scanning Electron Microscopy (SEM)

**Figure 6:**
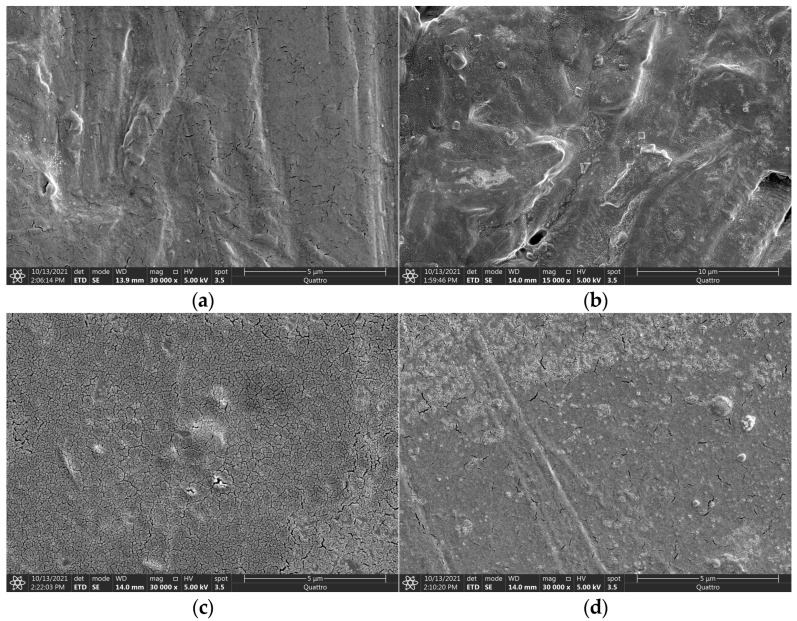
Scanning electron microscopy of wood composite samples at ×30,000 magnification: (**a**) HDPE-control sample, (**b**) HDPE-18 wt.% wood sample, (**c**) PP-control sample, and (**d**) PP-18 wt.% wood sample.

The main goal of incorporating wood fiber into a thermoplastic matrix (HDPE or PP) is to enhance the material’s mechanical integrity through reinforcement. The effectiveness of this reinforcement is evaluated using tensile property measurements. Achieving effective reinforcement relies on the thorough mixing of the wood filler with the polymer, which requires sufficiently low melt viscosity at the processing temperature. This enables the polymer to penetrate the porous structure of the wood and establish strong interfacial bonding. A robust interface between the wood fiber and polymer is essential for achieving desirable mechanical properties in composites. In this study, maleated polyethylene was used as a coupling agent to improve the compatibility between the hydrophilic wood fibers and the hydrophobic polymer. Scanning electron microscopy (SEM) images demonstrate an excellent dispersion of wood fibers within the polymer matrix, with no large aggregates observed in the analyzed samples. Selected micrographs are presented in Figure 6.

### 3.7. Gel Permeation Chromatography (GPC)

The mechanical stresses experienced during compounding and injection molding are anticipated to cause some degree of polymer composite degradation. This issue is particularly critical because processing must occur at lower temperatures to prevent the degradation of the wood fiber composites. While degradation affects both virgin polymers and polymer–wood fiber composites, it is anticipated to be more pronounced in the composites due to the increased friction from the wood filler.

Gel Permeation Chromatography (GPC) was used to analyze the polymer fraction of virgin polymers and composites with a low wood fiber amount (18 wt.%) before and after processing. The data in Table 3 reveal a reduction in the average molecular weight (Mn) for both PP and HDPE polymers as well as their wood–polymer composites. As expected, based on their chemical and structural differences, the PP showed a relatively higher degradation rate compared to the HDPE polymer. This is attributed to the generation of highly reactive tertiary carbon radicals during the mechanical and mechano-oxidative degradation of the PP polymer, in contrast to the less reactive secondary radicals generated by the HDPE polymer. The observed decreases in Mn values for the HDPE and PP composites were 15% and 12.6%, respectively, showing no significant difference at this wood fiber content. The polydispersity index (PDI) of the HDPE polymer decreased slightly upon mixing with wood fiber, while the PDI of the PP polymer suggested a broadening of the molecular weight distribution, likely due to the higher reactivity of the PP polymer. The molecular weights for composites with higher wood fiber content were not measured.

## 4. Conclusions

The durability of two sets of WPCs, one based on PP and another on HDPE, was investigated using both natural weathering under harsh desert exposure as well as laboratory-accelerated weathering. The average tensile strength of all composites as well as the respective controls decreased monotonously with both weathering exposures. Their tensile moduli increased with weathering indicating extensive crosslinking and possible increased crystallization during the weathering exposure. Changes in hardness with exposure also showed a similar decrease under both exposure regimens. The changes obtained compare well with those reported for WPCs using different wood fibers. An interesting correlation between hardness and tensile properties was obtained, but only for PP-based composites.

## Figures and Tables

**Table 1:** Change in average tensile properties of the wood–plastic composites of HDPE and PP after weathering exposures.

Outdoor Exposure Duration	Material	Tensile Strength (Mpa)		% Elongation at Break		Modulus of Elasticity (MPa)	
		Average	Std. Dev.	Average	Std. Dev.	Average	Std. Dev.
**Unexposed**	HDPE Control	23.67	0.23	1011.87	3.68	619.01	35.89
	HDPE-18% wood	29.35	0.79	24.53	3.53	1010.31	51.08
	HDPE-27% wood	33.05	0.84	13.75	1.52	1226.15	38.98
	HDPE-36% wood	35.36	0.79	9.45	0.90	1461.73	56.92
	PP Control	37.84	0.18	43.15	7.41	991.73	3.07
	PP-18% wood	38.42	0.38	9.52	0.57	1370.90	12.70
	PP-27% wood	38.73	0.30	6.93	0.36	1553.33	42.83
	PP-36% wood	38.77	0.63	5.87	0.21	1712.30	14.51
**6 Months**	HDPE Control	4.67	0.99	5.34	7.01	969.82	39.14
	HDPE-18% wood	28.50	0.99	13.78	1.60	1051.20	44.41
	HDPE-27% wood	31.97	0.76	11.05	0.91	1264.55	45.03
	HDPE-36% wood	34.47	0.60	8.05	0.34	1494.55	36.63
	PP Control	16.70	1.75	2.40	0.39	1052.09	5.26
	PP-18% wood	31.83	0.35	6.93	0.40	1295.30	28.43
	PP-27% wood	33.38	0.18	5.74	0.15	1492.23	22.43
	PP-36% wood	33.38	0.24	4.92	0.17	1651.79	27.03
**12 Months**	HDPE Control	4.41	0.99	1.55	0.55	776.78	20.32
	HDPE-18% wood	25.20	0.85	11.70	0.53	1004.87	32.70
	HDPE-27% wood	29.82	0.49	8.70	0.80	1244.91	28.31
	HDPE-36% wood	32.35	0.55	7.86	0.25	1443.60	40.78
	PP Control	9.57	0.20	1.55	0.06	833.50	21.66
	PP-18% wood	28.02	0.53	7.18	0.57	1132.39	25.34
	PP-27% wood	30.20	0.21	5.69	0.18	1378.63	22.98
	PP-36% wood	30.34	0.44	4.60	0.23	1544.98	31.86
**18 Months**	HDPE Control	4.55	0.55	1.09	0.15	781.58	41.13
	HDPE-18% wood	24.23	0.94	9.98	0.92	1000.21	27.54
	HDPE-27% wood	28.62	0.61	9.39	0.67	1204.85	29.77
	HDPE-36% wood	31.11	0.40	7.78	0.27	1376.91	24.97
	PP Control	3.63	0.45	1.42	0.69	630.65	32.97
	PP-18% wood	26.30	0.20	6.53	0.34	1063.13	22.03
	PP-27% wood	28.75	0.46	5.63	0.30	1275.50	50.53
	PP-36% wood	30.00	0.24	4.67	0.29	1483.51	20.35
**Accelerated Exposure Time/Energy**	**Material**	**Tensile Strength (Mpa)**		**% elongation at break**		**Modulus of Elasticity (MPa)**	
		**Average**	**Std. Dev.**	**Average**	**Std. Dev.**	**Average**	**Std. Dev.**
**214 h Exposure Energy = 276.8 kJ/sq.mt.**	HDPE Control	15.27	0.42	6.37	2.17	793.17	77.97
	HDPE-18% wood	29.38	0.71	15.15	1.01	1090.50	17.23
	HDPE-27% wood	33.47	0.26	10.40	0.76	1361.87	18.56
	HDPE-36% wood	34.52	1.44	8.16	0.56	1488.11	98.97
	PP Control	33.05	-	8.38	-	1034.57	-
	PP-18% wood	35.38	0.45	7.59	0.38	1402.82	31.60
	PP-27% wood	36.33	0.22	5.65	0.26	1613.51	34.87
	PP-36% wood	34.91	0.58	4.60	0.28	1761.16	16.45
**483 h Exposure Energy = 624.9 kJ/sq.mt.**	HDPE Control	14.14	0.79	11.14	1.62	628.23	13.99
	HDPE-18% wood	28.44	0.48	13.50	1.46	1078.97	19.89
	HDPE-27% wood	31.79	0.86	10.00	0.60	1297.29	40.10
	HDPE-36% wood	35.19	0.89	7.69	0.39	1566.84	54.39
	PP Control	23.19	-	7.12	-	853.28	-
	PP-18% wood	33.01	0.19	6.33	0.12	1363.88	24.19
	PP-27% wood	34.06	0.01	5.58	0.22	1535.70	3.10
	PP-36% wood	33.94	0.27	4.53	0.19	1777.21	61.82
**723 h Exposure Energy = 945 kJ/sq.mt.**	HDPE Control	14.45	0.20	11.37	1.68	558.04	21.26
	HDPE-18% wood	28.58	0.47	11.41	0.55	1119.73	12.33
	HDPE-27% wood	32.38	0.17	8.85	0.09	1337.09	9.03
	HDPE-36% wood	35.05	1.57	7.42	0.14	1549.66	96.14
	PP Control	14.34	-	5.92	-	645.15	-
	PP-18% wood	30.35	0.69	6.07	0.32	1253.94	23.91
	PP-27% wood	32.09	0.23	5.27	0.36	1488.93	58.20
	PP-36% wood	32.28	0.31	4.36	0.11	1665.32	11.43
**1180 h Exposure Energy = 1514.6 kJ/sq.mt.**	HDPE Control	12.35	-	9.15	-	479.71	-
	HDPE-18% wood	28.22	1.39	10.37	0.56	1093.23	56.28
	HDPE-27% wood	32.31	1.19	8.43	0.65	1360.65	71.09
	HDPE-36% wood	35.31	0.91	6.56	0.19	1579.18	49.82
	PP Control	4.68	-	4.58	-	353.50	-
	PP-18% wood	28.35	0.46	6.12	0.13	1135.02	35.05
	PP-27% wood	30.97	0.33	5.15	0.35	1451.05	23.63
	PP-36% wood	31.13	0.35	4.14	0.14	1661.96	54.21

**Table 2:** Change in average Shore D hardness values of wood–plastic composites of HDPE and PP after weathering exposures.

Outdoor Exposure Duration	Average Hardness Value [Std. Dev.]			
	HDPE Control	HDPE-18% Wood	HDPE-27% Wood	HDPE-36% Wood
Unexposed	63.2 [0.1]	65.8 [0.2]	66.7 [0.4]	68.3 [0.6]
6 months	56.9 [2.1]	67.5 [0.3]	66.4 [0.4]	68.5 [2.0]
12 months	56.4 [2.3]	63.4 [0.8]	63.6 [1.2]	67.6 [0.2]
18 months	56.3 [1.7]	62.9 [1.7]	61.2 [0.7]	64.4 [0.7]
**Outdoor Exposure Duration**	**Average Hardness Value [Std. Dev.]**			
	**PP Control**	**PP-18% wood**	**PP-27% wood**	**PP-36% wood**
Unexposed	73.9 [0.8]	73.4 [1.0]	73.3 [0.6]	73.9 [1.1]
6 months	67.8 [1.1]	68.0 [1.7]	69.8 [0.8]	68.1 [1.3]
12 months	68.1 [0.6]	67.4 [1.2]	68.1 [1.2]	69.5 [0.8]
18 months	60.1 [2.1]	60.8 [1.9]	65.3 [1.5]	67.8 [2.1]
**Accelerated Exposure Duration**	**Average Hardness Value [Std. Dev.]**			
	**HDPE Control**	**HDPE-18% wood**	**HDPE-27% wood**	**HDPE-36% wood**
214 h	63.2 [1.2]	64.4 [0.8]	66.0 [1.3]	68.8 [1.1]
483 h	54.6 [2.6]	65.1 [1.4]	66.0 [1.3]	67.6 [2.1]
723 h	53.9 [1.0]	61.7 [3.0]	64.3 [1.6]	67.5 [1.0]
1180 h	47.5 [2.8]	60.1 [1.1]	65.5 [0.7]	67.1 [1.5]
**Accelerated Exposure Duration**	**Average Hardness Value [Std. Dev.]**			
	**PP Control**	**PP-18% wood**	**PP-27% wood**	**PP-36% wood**
214 h	72.3 [1.2]	70.8 [1.0]	71.8 [1.4]	73.0 [0.8]
483 h	67.0 [0.7]	69.7 [1.5]	72.0 [1.3]	71.0 [1.7]
723 h	62.6 [1.5]	66.6 [1.9]	69.3 [1.1]	72.4 [1.0]
1180 h	54.8 [1.0]	65.4 [3.5]	68.2 [2.4]	70.3 [0.8]

**Table 3:** Changes in the average molecular weights (g/mol) of wood–plastic composites of HDPE and PP after outdoor weathering exposure.

Exposure Duration	Material	Mw	Mn	PDI
Unexposed	HDPE Control	78,428	15,208	5.16
	HDPE-18% wood	61,839	12,871	4.81
6 Months	HDPE Control	26,680	7481	3.57
	HDPE-18% wood	69,140	15,891	4.35
12 Months	HDPE Control	23,019	5929	3.88
	HDPE-18% wood	72,990	14,369	5.08
18 Months	HDPE Control	23,282	5426	4.29
	HDPE-18% wood	60,495	15,550	3.89
**Exposure Duration**	**Material**	**Mw**	**Mn**	**PDI**
Unexposed	PP Control	194,387	37,189	5.23
	PP-18% wood	190,151	32,495	5.85
6 Months	PP Control	31,292	7899	3.96
	PP-18% wood	189,532	30,840	6.15
12 Months	PP Control	7398	3154	2.35
	PP-18% wood	167,423	26,657	6.28
18 Months	PP Control	5975	2906	2.06
	PP-18% wood	155,017	28,854	5.37

## Data Availability

The original contributions presented in this study are included in the article; further inquiries can be directed to the corresponding author.

## References

[1] IEA (2020). World Energy Outlook 2020.

[2] Balu R., Dutta N.K., Roy Choudhury N. (2022). Plastic Waste Upcycling: A Sustainable Solution for Waste Management, Product Development, and Circular Economy. Polymers.

[3] Kassab A., Al Nabhani D., Mohanty P., Pannier C., Ayoub G.Y. (2023). Advancing Plastic Recycling: Challenges and Opportunities in the Integration of 3D Printing and Distributed Recycling for a Circular Economy. Polymers.

[4] Gamboa C. (2020). Towards Zero-Carbon Building In Climate 2020.

[5] Rowell R.M. (2021). Understanding Wood Surface Chemistry and Approaches to Modification: A Review. Polymers.

[6] Kučerová V., Hrčka R., Hýrošová T. (2022). Relation of Chemical Composition and Colour of Spruce Wood. Polymers.

[7] Kumar B.G., Singh R.P., Nakamura T. (2002). Degradation of Carbon Fiber-Reinforced Epoxy Composites by Ultraviolet Radiation and Condensation. J. Compos. Mater..

[8] Broda M., Popescu C.M., Curling S.F., Timpu D.I., Ormondroyd G.A. (2022). Effects of Biological and Chemical Degradation on the Properties of Scots Pine Wood-Part I: Chemical Composition and Microstructure of the Cell Wall. Materials.

[9] Friedrich D. (2021). Thermoplastic Moulding of Wood-Polymer Composites (WPC): A review on physical and mechanical behaviour under hot-pressing technique. Compos. Struct..

[10] Olonisakin K., He S., Yang Y., Wang H., Li R., Yang W. (2022). Influence of stacking sequence on mechanical properties and moisture absorption of epoxy-based woven flax and basalt fabric hybrid composites. Sustain. Struct..

[11] Khan M.M.H., Deviatkin I., Havukainen J., Horttanainen M. (2021). Environmental impacts of wooden, plastic, and wood-polymer composite pallet: A life cycle assessment approach. Int. J. Life Cycle Assess..

[12] Muller U., Veigel S. (2022). The strength and stiffness of oriented wood and cellulose-fiber materials: A review. Prog. Mater. Sci..

[13] Smith P.M., Wolcott M.P. (2006). Opportunities for Wood/Natural Fiber-Plastic Composites in Residential and Industrial applications. For. Prod. J..

[14] Gardner D., Han Y., Song W. Wood plastic composites technology trends. Proceedings of the 51st International Convention of Society of Wood.

[15] Panthapulakkal S., Zereshkian A., Saim M. (2006). Preparation and characterization of wheat straw fibers for reinforcing application in injection molded thermoplastic composites. Bioresour. Technol..

[16] Zhang W., Yao X., Khanal S., Xu S. (2018). A novel surface treatment for bamboo flour and its effect on the dimensional stability and mechanical properties of high-density polyethylene/bamboo flour composites. Constr. Build. Mater..

[17] Oladejo K.O., Omoniyi T.E. (2017). Dimensional Stability and Mechanical Properties of Wood Plastic Composites Products from Sawdust of Anogeissus leiocarpus (Avin) with Recycled Polyethylene Teraphthalate (PET) Chips. Eur. J. Appl. Eng. Sci. Res..

[18] Najafi S.K. (2013). Use of Recycled Plastics in Wood Plastic Composites—A Review. Waste Manag..

[19] Binhussain M.A., El-Tonsy M.M. (2013). Palm leave and plastic waste wood composite for out-door structures. Constr. Build. Mater..

[20] Ratanawilai T., Taneerat K. (2018). Alternative polymeric matrices for wood-plastic composites: Effects on mechanical properties and resistance to natural weathering. Constr. Build. Mater..

[21] Wu M., Zhao M., Chang G., Hu X., Guo Q. (2019). A composite obtained from waste automotive plastics and sugarcane skin flour: Mechanical properties and thermo-chemical analysis. Powder Technol..

[22] Dauletbek A., Li H., Xiong Z., Lorenzo R. (2021). A review of mechanical behavior of structural laminated bamboo lumber. Sustain. Struct..

[23] Grubbström G., Holmgren A., Oksman K. (2010). Silane-crosslinking of recycled low-density polyethylene/wood composites—ScienceDirect. Compos. Part A Appl. Sci. Manuf..

[24] Carus M., Eder A. (2015). WPC and NFC market trends, bioplastics. Magazine.

[25] Nevin G.K., Ayse A. (2011). Effects of maleated polypropylene on the morphology, thermal and mechanical properties of short carbon fiber reinforced polypropylene composites. Mater. Des..

[26] Pickering K., Efendy M.A., Le T. (2016). A review of recent developments in natural fibre composites and their mechanical performance. Compos. Part A Appl. Sci. Manuf..

[27] Razavi-Nouri M., Tayefi M., Sabet A. (2017). Morphology development and thermal degradation of dynamically cured ethylene-octene copolymer/organoclay nanocomposites. Thermochim. Acta.

[28] Li H., Zhang Z., Song K., Lee S., Chun S.-J., Zhou D., Wu Q. (2014). Effect of durability treatment on ultraviolet resistance, strength, and surface wettability of wood plastic composite. BioResources.

[29] Stark N.M. Considerations in the weathering of wood-plastic composites. Proceedings of the 3rd Wood Fibre Polymer Composites International Symposium: Innovative Sustainable Materials Applied to Building and Furniture.

[30] Teacă C.-A., Roşu D., Bodîrlău R., Roşu L. (2013). Structural changes in wood under artificial UV light irradiation determined by FTIR spectroscopy and color measurements—A brief review. BioResources.

[31] Doğan M. (2021). Ultraviolet light accelerates the degradation of polyethylene plastics. Microsc. Res. Tech..

[32] Hon D.N.S. (2001). Wood and Cellulosic Chemistry.

[33] Selden R., Nystrom B., Langstrom R. (2004). UV aging of polypropylene/wood fiber composites. Polym. Compos..

[34] Beg M., Pickering K. (2008). Accelerated weathering of unbleached kraft wood fibre reinforced polypropylene composites. Polym. Degrad. Stab..

[35] Mantia F., Morreale M. (2008). Accelerated weathering of polypropylene/wood flour composites. Polym. Degrad. Stab..

[36] Soccalingame L., Rerrin D., Benezet J. (2015). Reprocessing of artificial UV weathered wood flour reinforced polypropylene composites. Polym. Degrad. Stab..

[37] Phiriyawirut M., Seenpung P., Calermboom S. (2008). Isostatic polypropylene/wood sawdust composite: Effects of natural weathering, water immersion and Ga, a-Ray Irradiation on Mechanical Properties. Macromol. Symp..

[38] Darabi P., Karimi A.N., Mirshokraie S.A., Thévenon M.F. Lignin blocking effects on weathering process of wood plastic composites. Proceedings of the 41st Annual Meeting of the International Research Group on Wood Protection.

[39] Lei Z., Liu J., Zhang Z., Zhao X., Li Q. (2023). Study on Preparation and Properties of Anti-Ultraviolet Aging Wood-Plastic Composites. Wood Res..

[40] (2022). Wood Plastic Composites Market Size. Share & Trend Analysis Report by Product (Polyethylene, Polypropylene), by Application (Automotive Components), by Region, and Segment Forecasts, 2022–2030.

[41] Redhwi H.H., Siddiqui M.N., Andrady A.L., Furquan S.A., Hussain S. (2023). Durability of High-Density Polyethylene (HDPE) and Polypropylene (PP) based Wood-Plastic Composites Part 1: Mechanical Properties of the Composite Materials. J. Comps. Sci..

[42] Umar A.H., Zainudin E.S., Sapuan S.M. (2012). Effect of Accelerated Weathering n Tensile Properties of Kenaf Reinforced High-Density Polyethylene Composites. J. Mech. Eng. Sci..

[43] Hung K.C., Chang W.C., Xu J.W., Wu T.L., Wu J.H. (2021). Comparison of the Physico-Mechanical and Weathering Properties of Wood-Plastic Composites Made of Wood Fibers from Discarded Parts of Pomelo Trees and Polypropylene. Polymers.

[44] Gunjal J., Aggarwal P., Chauhan S. (2020). Changes in colour and mechanical properties of wood polypropylene composites on natural weathering. Maderas. Cienc. Tecnol..

[45] López-Naranjo E.J., Alzate-Gaviria L.M., Hernández-Zárate G., Reyes-Trujeque J., Cruz-Estrada R.H. (2014). Effect of accelerated weathering and termite attack on the tensile properties and aesthetics of recycled HDPE-pinewood composites. J. Thermoplast. Compos. Mater..

[46] Stark N.M., Matuana L.M., Clemons C.M. (2004). Effect of processing method on surface and weathering characteristics of wood-flour/HDPE composites. J. Appl. Polym. Sci..

[47] Xanthopoulou E., Chrysafi I., Polychronidis P., Zamboulis A., Bikiaris D.N. (2023). Evaluation of Eco-Friendly Hemp-Fiber-Reinforced Recycled HDPE Composites. J. Compos. Sci..

[48] Li J., Huo R., Liu W., Fang H., Jiang L., Zhou D. (2022). Mechanical properties of PVC-based wood–plastic composites effected by temperature. Front. Mater..

[49] Grause G., Chien M.F., Inoue C. (2020). Changes during the weathering of polyolefins. Polym. Degrad. Stab..

[50] Oluwoye I., Altarawneh M., Gore J., Dlugogorski B.Z. (2015). Oxidation of crystalline polyethylene. Combust. Flame.

[51] Kanbayashi T., Matsunaga M., Kobayashi M. (2021). Cellular-level chemical changes in Japanese beech (Fagus crenata Blume) during artificial weathering. J. Holzforsch..

[52] Andrady A.L., Barnes P.W., Bornman J.F., Gouin T., Madronich S., White C.C., Zepp R.G., Jansen M.A.K. (2022). Oxidation and fragmentation of plastics in a changing environment; from UV-radiation to biological degradation. Sci. Total Environ..

[53] Homkhiew C., Ratanawilai T., Thongruang W. (2014). Effects of natural weathering on the properties of recycled polypropylene composites reinforced with rubberwood flour. Ind. Crops Prod..

[54] Nukala S.G., Kong I., Kakarla A.B., Kong W., Kong W. (2022). Development of wood polymer composites from recycled wood and plastic waste: Thermal and mechanical properties. J. Compos. Sci..

[55] Srivabut C., Homkhiew C., Rawangwong S., Boonchouytan W. (2022). Possibility of using municipal solid waste for manufacturing wood plastic composites: Effects of natural weathering, wood waste types, and contents. J. Mater. Cycles. Waste. Manag..

[56] Srivabut C., Khamtree S., Homkhiew C., Ratanawilai T., Rawangwong S. (2023). Comparative effects of different coastal weathering on the thermal, physical, and mechanical properties of rubberwood–latex sludge flour reinforced with polypropylene hybrid composites. Compos. Part C Open Access.

